# Cervical lateral mass screw fixation without fluoroscopic control: analysis of risk factors for complications associated with screw insertion

**DOI:** 10.1007/s00402-012-1507-6

**Published:** 2012-03-30

**Authors:** Shinichi Inoue, Tokuhide Moriyama, Toshiya Tachibana, Fumiaki Okada, Keishi Maruo, Yutaka Horinouchi, Shinichi Yoshiya

**Affiliations:** Department of Orthopaedic Surgery, Hyogo College of Medicine, 1-1 Mukogawa-cho, Nishinomiya, Hyogo 663-8501 Japan

**Keywords:** Lateral mass, Cervical spine, Posterior fixation, Complications

## Abstract

**Objective:**

To examine the outcome of cervical lateral mass screw fixation focusing on analysis of the risk factors for screw-related complications.

**Methods:**

Ninety-four patients who underwent posterior cervical fixation with a total of 457 lateral mass screws were included in the study. The lateral mass screws were placed using a modified Magerl method. Computed tomographic (CT) images were taken in the early postoperative period in all patients, and the screw trajectory angle was measured on both axial and sagittal plane images.

**Results:**

In the postoperative CT analysis for the screw trajectory, 56.5 % of the screws were directed within the acceptable range (within 21–40° on both axial and sagittal planes). As intraoperative screw-associated complications, 9.6 % of the screws were found to contact with or breach the vertebral artery foramen. In this group, the screw trajectory angle on axial plane was significantly lower than in the group without contact. Facet violation was observed in 13 screws (2.8 %). This complication was associated with a significantly lower trajectory angles in the sagittal plane, predominantly at C6 level (69.2 %). In the patient chart review, no serious neurovascular injuries were documented.

**Conclusions:**

In the analysis of potential risk factors for violation of the VA foramen as well as FV during screw insertion, the former incidence was significantly related to the screw trajectory angle (lack of lateral angulation) in the axial plane, while the latter incidence was related to a poor screw trajectory angle in the sagittal plane.

## Introduction

Posterior cervical fixation is a commonly selected procedure in the surgical management of the unstable cervical spine caused by trauma, and other morbidities such as degenerative disorders, neoplasms, rheumatoid arthritis, and destructive spondyloarthopthy. For fixation, the application of wire between the spinal processes was the first technique described in the literature [1]. Subsequently, various screw fixation techniques using the lateral mass screw, pedicle screw and transarticular screw have been introduced, and clinical experiences with these techniques have been reported [2–7].

Among those techniques, the pedicle screw is predominantly used in Japan. Previous biomechanical experiments performed for this fixation method have shown its superior strength compared to other techniques [8], and favorable clinical outcomes have also been reported. However, the potential risk of vertebral artery (VA) injury is a concern with this technique [9, 10]. To avoid this devastating complication, use of a navigation system and various other imaging aids has been reported [11–13].

Another option for posterior cervical fixation is the use of a screw applied to the lateral mass as an internal fixation device (lateral mass screw fixation). Roy-Camille et al. [2] initially proposed this procedure in the 1980s. As a result, this operative procedure was further developed and promoted by Anderson, An, and Magerl [3–5]. There have been several articles claiming that lateral mass screwing is simple, safe, and effective compared to other fixation techniques. An additional advantage of lateral mass screwing is the elimination of the need for intraoperative fluoroscopic control [14, 15]. As a result, this fixation method is presently one of the most prevailing procedures in posterior cervical fixation throughout the world [14–22].

However, there are complications in the use of lateral mass screwing, and VA, facet violation (FV), nerve root injuries, and lateral mass fracture are listed as potential intraoperative complications associated with screw insertion [16]. Among the risk factors for these complications, an inappropriate screw trajectory has been pinpointed as a critical factor [23–30]. In cadaveric experiments simulating the surgical procedure, Heller et al. [23], Seybold et al. [29], and Barrey et al. [27] showed a correlation between the risk of these complications and an inappropriate screw trajectory angle. In the analysis of clinical results, Graham and Roche claimed that the screw positioning is the main factor leading to those complications [14, 15]. To date, however, the significance of the screw trajectory angle as related to the potential risk for injury to the adjacent structures has not been clarified.

In this study, the screw trajectory angle was evaluated on CT images taken in the early postoperative period. We hypothesized that there is a correlation between the screw trajectory angle and the potential risk for screw-related complications. The purpose of the present study was to review our clinical experience with this fixation technique, focusing on the analysis of risk factors for complications associated with cervical lateral mass screwing.

## Materials and methods

### Subjects

The design of this study is a retrospective clinical review of our patient population who underwent cervical lateral mass screw fixation. One hundred and seven patients underwent this procedure at our institute from 2000 to 2010. Among these patients, CT images and clinical records in the early postoperative period were available in 94 patients (49 men and 45 women) and this patient population constituted the basis of the study. Screw placement and trajectory were assessed on the CT images taken within 3 weeks, while clinical findings indicating screw-related complications were reviewed in the patient’s chart within 1 month after surgery. The screws inserted at C7 were excluded from the analysis, since the pedicle screw was the device of choice at this level. In addition, C1 and C2 levels were not included in the analysis since lateral mass screw fixation was not applied to these levels in our practice during the study period. In total, 457 lateral mass screws were used and subject to analysis. The screws were inserted using the modified Magerl method. The average age at surgery was 56.8 year (range 15–86 years). Detailed information of the patient demographics such as body weight, height, and BMI is presented in Table 1. The preoperative diagnoses were degenerative disorders (cervical spondylotic myelopathy and ossification of the posterior longitudinal ligament; OPLL) in 29, trauma in 24, rheumatoid arthritis in 15, cerebral palsy in 4, destructive spondyloarthropathy in 6, tumor in 11, and other lesions in 5 patients. Various instrumentation systems were used for fixation including Axis system (Sofamor Danek, Memphis), Olerud system (Anatomica, Sweden) and OASYS system (Stryker Spine, France) for 15, 26, and 53 patients, respectively.

**Table 1:** Patient demographics

Average age (years)	56.8 ± 16.2 (15–86)
Gender (female:male)	45:49
Height (cm)	159.5 ± 9.5 (138–181)
Body weight (kg)	58.7 ± 14.7 (30–94)
Body mass index	22.9 ± 4.8 (15.0–37.8)

### Surgical procedure (Fig. 1)

During surgery, the patient is placed in the prone position after endotracheal intubation. Alignment of the cervical spine is maintained at neutral during the procedure using the three-pin skull fixation. We only use fluoroscopy to confirm the alignment of the cervical spine preoperatively. A standard midline posterior approach to the cervical spine is used. Posterior elements are fully exposed, extending to the lateral edges of the lateral mass and the facet joint at each fusion level. The facet joints to be fused are decorticated, while care is taken to protect the facet joint above and below the instrumented levels. The entry point was located 1 mm medial to the mid-point of the lateral mass. An awl was used to create the starting hole. The angle of screw trajectory was directed approximately 30° laterally and superiorly (parallel to the facet joint), which is a modification of Magerl’s proposal. Since the superolateral quadrant is regarded as the “safe zone” [24], this region is used as an imaginary target during the drilling. Drilling and tapping are directed toward the superior lateral ventral corner without the help of fluoroscopic guidance. In most cases, fixation is performed after completing decompression procedures such as laminectomy or laminoplasty. Drilling is started at a depth of 14 mm, and further advanced, when feasible, until bicortical screw purchase is achieved.

**Fig. 1:**
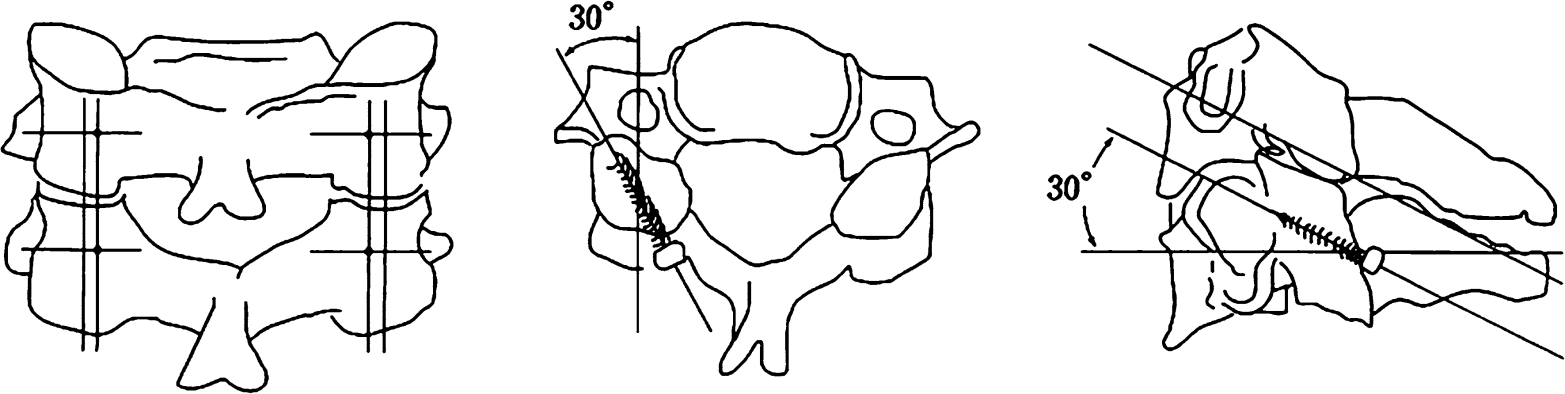
Schematic presentation of the modified Magerl technique employed in our clinical practice. The entry point in this procedure is located 1 mm medial to the midpoint of the lateral mass. The screw is directed approximately 30° both laterally and superiorly (parallel to the facet joint)

### Postoperative management

Patients are instructed to wear a semi-rigid collar or a soft collar for at least 8 weeks after surgery except for patients with cerebral palsy. For patients with cerebral palsy, we apply a Halo-vest for at least 8 weeks followed by additional use of a semi-rigid collar for an additional 4 weeks.

### CT evaluation for screw trajectory

**Fig. 2:**
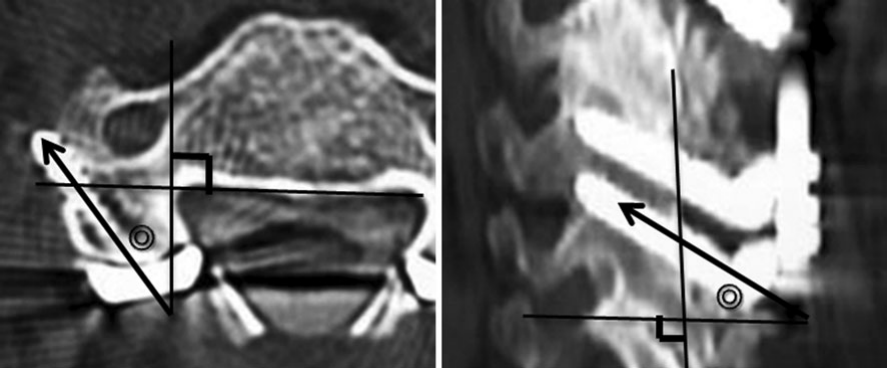
Determination of orientation of screw trajectory in the image analysis. Axial plane: the angle between the axial screw trajectory and the line perpendicular to the tangential line behind the vertebral body. Sagittal plane: the angle between the sagittal trajectory and the line perpendicular to the tangential line behind the lateral mass

For all the included patients, CT examinations were performed within 3 weeks after the surgery. The screw trajectory angle was measured on both axial and sagittal planes following the method described by Seybold et al. [29]. Among the serial CT images taken with a slice thickness of 3 mm, the axial slices including the VA foramen and sagittal slices including the facet joint were selected for each of the cervical levels. Violation of the VA foramen as well as the facet joint by the screw was assessed on those images. Screw trajectory was measured using a ruler and a goniometer on the printed CT images showing the screw as well as the bony landmarks (Fig. 2). The acceptable range of the screw trajectory angle was defined within 21–40° on both axial and sagittal planes.

### Complications associated with screwing

The spatial relationship between the screw and the VA foramen was evaluated on axial plane CT images. Based on the location of the screw tip in relation to the edge of the VA foramen, the screws subjected to the analysis were divided into two groups as follows. When the screws were shown to contact with or violate the VA foramen these screws were classified as contacting and allocated to the contact group. By contrast, the screws without any contact with the edge of the foramen were classified as not contacting and allocated to the non-contact group. The presence of FV was also assessed on CT images. Based on the location of the screw in relation to the facet joint, screws were divided into two groups (non-FV and FV groups). In addition, occurrence of intraopertive lateral mass fracture was assessed on the postoperative CT images.

Regarding occurrence of intraoperative VA injury, the operative record was reviewed for description of profuse arterial bleeding. Description of any symptoms and signs indicating neurovascular injuries such as sensory or motor deficit, and visual impairment was reviewed in the patient chart. The data collection based on the chart review was limited to the descriptions during the initial month after the index surgery.

To evaluate the potential risk of these complications, the length and trajectory angle of each screw were measured on the CT images and compared between the groups with and without the complications.

### Statistical analysis

All numerical results were presented as mean ± SD. Differences between the groups were compared using the unpaired t-test and Fisher`s exact probability test. Differences between each of the fixation levels were analyzed with one-way ANOVA followed by Fisher`s PLSD test. A difference was accepted as significant if the probability was less than 5 % (*P* < 0.05).

## Results

### CT evaluation for screw length and trajectory

A total of 457 lateral mass screws comprising 89 at C3, 140 at C4, 138 at C5, and 90 screws at C6 level. The mean screw length at each level was 16.2 ± 1.7 mm at C3, 16.4 ± 1.5 mm at C4, 16.3 ± 1.7 mm at C5 and 16.2 ± 1.7 mm at C6. There was no significant difference in screw length among the levels (*P* = 0.8081).

The mean screw trajectory angles on the axial and sagittal planes were 25.9° ± 6.4° and 29.0° ± 9.3°, respectively (Table 2). The measured angles were within the acceptable range both on axial and sagittal planes (between 21° and 40°) in 258 of 457 screws (56.5 %). When the screw trajectory angle was compared among the levels, significant differences were detected only on the sagittal plane.

**Table 2:** Average screw trajectory angles on the axial and sagittal planes at each level

	C3	C4	C5	C6	All	*P* value
Axial angle (°)	25.2 ± 7.4	26.2 ± 6.1	26.6 ± 6.0	24.9 ± 6.3	25.9 ± 6.4	0.1600
Sagittal angle (°)	31.0 ± 8.8	29.9 ± 8.8	28.6 ± 9.6	26.2 ± 9.7	29.0 ± 9.3	0.0034

### **Contacted or violation of the vertebral artery (VA) foramen** (Table 3)

**Fig. 3:**
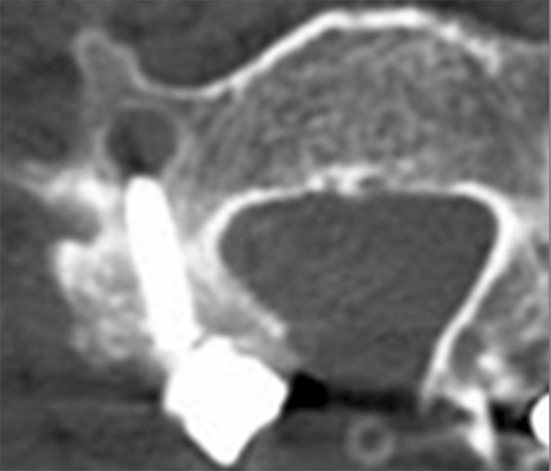
Violation of the edge of VA foramen by the left lateral mass screw is identified. The screw trajectory angle in the axial plane is 5°

Analysis of the axial image at each level revealed contact of the screw with the edge of the VA foramen in 8.5 % (39 of 457) of the screws. Moreover, 5 screws (1.1 %) were observed to violate the edge of the foramen **(**Fig. 3). In total, 44 screws (9.6 %) were classified as the contact group, while no contact or violation was observed on CT images in the remaining 413 group 2 screws (90.4 %). In the analysis of the factors related to the contact or violation of the screw, it was shown that the axial trajectory angle in the contact group (18.5°) was significantly lower than the angle in the non-contact group (26.7°) with a statistical significance (*P* < 0.0001). When the axial trajectory angle in the contact group was compared among the levels, no difference was detected. In the comparison of the sagittal projection angle and screw length between the contact and non-contact groups, no significant difference was observed.

**Table 3:** Average screw length and angles and spatial relationship with the vertebral foramen

	Non-contact group	Contact group	*P* value
	413 screws	44 screws	
Screw length (mm)	16.2 ± 1.6	16.7 ± 1.4	0.0605
Axial angle (°)	26.7 ± 5.9	18.5 ± 6.8	<0.0001
Sagittal angle (°)	29.2 ± 9.5	26.7 ± 8.0	0.0904

### Facet violation (FV) (Table 4)

**Fig. 4:**
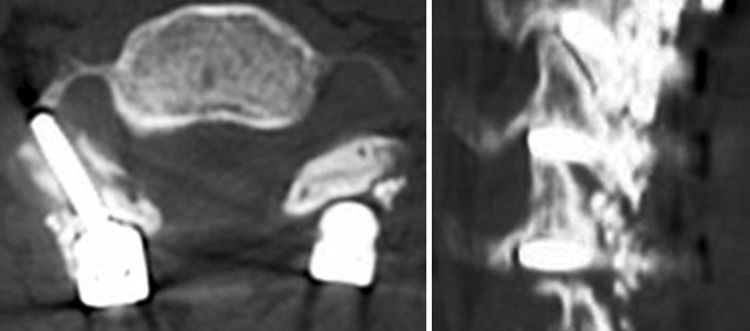
Facet violations at the C5/6 facet and the C6/7 facet by the lateral mass screw are identified

In the analysis of the CT images, FV were identified for 13 screws **(**Fig. 4). The screw trajectory angle on the sagittal plane in this FV group (12.3°) was significantly lower than the corresponding angle in the non-FV group (29.5°) with statistical significance (*P* < 0.0001). When the sagittal projection angle in the contact group was compared among the levels, no difference was detected. In the comparison of the axial projection angle and screw length between the FV (24.2° and 15.7 mm, respectively) and non-FV groups (26.0° and 16.3 mm, respectively), no difference was demonstrated. With regard to the incidence of this complication among cervical levels, 69.2 % of the facet violation was detected at the C6 level with a significantly higher incidence compared to the other levels (*P* < 0.0001). No significant correlation between the occurrences of violation of the VA foramen and the FV was detected.

**Table 4:** Average screw length and angle in FV and non-FV groups

	Non-FV group	FV group	*P* value
	444 screws	13 screws	
Screw length (mm)	16.3 ± 1.6	15.7 ± 1.1	0.1810
Axial angle (°)	26.0 ± 6.4	24.2 ± 6.4	0.3190
Sagittal angle (°)	29.5 ± 12.3	12.3 ± 8.0	<0.0001

### Lateral mass fracture

Intraoperative lateral mass fractures were identified in 18 of the 471 lateral masses (3.8 %). When this complication was encountered during surgery, the screw was reinserted with a different trajectory angle in four lateral masses. In the remaining cases, screw reinsertion at the same level was deemed impossible and the corresponding site was skipped for screwing. Therefore, the relationship between the occurrence of this complication and the screw trajectory was not analyzed in this group.

### Chart review for postoperative course

No description indicating intraoperative injury to neurovascular structures was identified in the review of the patients’ chart. For early postoperative complications, surgical site infection and postoperative C5 root palsy occurred in 1 case (1.7 %) and 5 cases (5.3 %), respectively. Revision of the lateral mass screw was not required in any case.

## Discussion

In the present study, CT examination during the early postoperative period enabled accurate analysis of direction and depth of the screw on both axial and sagittal planes. Consequently, several findings of interest were demonstrated showing the relationship between the screw trajectory and screw-related complications.

In our clinical experiences, the precision of lateral mass screwing during fluoroscopic images was not high, because the rate of acceptable trajectory angle (between 21° and 40° on both axial and sagittal planes) was only 56.5 % (258/457 screws). Heller et al. [23] assessed the screw trajectory on the lateral radiograph (Magerl technique) in a cadaveric study, and showed that 58.5 % of the screws were within the intended zone in their grading system. Moreover, Graham et al. [14] described that screws with acceptable direction on both axial and sagittal planes were only 28.7 % in their clinical experiences, while no major neurovascular injuries were encountered in their series. Thus, achievement of accurate and consistent screw trajectory is still an issue to be pursued.

There remains a potential risk of VA injury in this technique, and two conclusions of note can be drawn. First, although the actual incidence of major vascular injury is very low (no cases in this series), close contact or even violation of the VA foramen by the screw can be present with considerable incidence (9.6 % in this series). This situation raises the possibility of vascular injury. Second, the low axial screw trajectory (lack of lateral angulation) was strongly correlated with this potential complication. In a cadaveric study, Seybold et al. [29] reported that a risk of VA injury was related to the axial deviation angle. Ebraheim et al. [30] performed an anatomic study and found that both Roy-Camille technique and Magerl technique could damage the vertebral artery unless a minimal 15° lateral angulation was maintained during drilling. In another paper, the same authors further reported the spatial relationship between the VA foramen and the posterior midpoint of the lateral mass was different between C3–5 and C6 with the VA foramen located directly in front of the posterior midpoint of the lateral mass at C6 [31].

FV is another screw-related complication reported in the literature, and this complication was encountered in 13 screws (2.8 %) in this series. Heller et al. [23] reported that the risk of FV was higher in Roy-Camille technique (22.5 %) than Magerl technique (2.4 %), while Barrey et al. [27] reported that facet violation occurred in 4 of 80 lateral mass screws (5.0 %) with the use of Magerl technique. To analyze the risk factors related to occurrence of FV, various parameters in the screw trajectory were statistically examined to discover whether there is any difference between the FV and non-FV groups. We found that a low screw trajectory angle in the sagittal plane was strongly correlated with the occurrence of this complication, while the trajectory angle on the axial plane and the screw length did not influence its incidence. Seybold et al. [29] also reported that the risk of FV was higher for the screw with a lower trajectory angle in the sagittal plane. Another characteristic finding in the present study is the considerably high incidence of this complication at the C6 level (69.2 %). This tendency is thought to be due to morphologic characteristics of this level. Barrey et al. [27] described that the sagittal safety angle became narrowest at C6. As claimed by Ebraheim et al. [25], violation of the inferior articular facet of the most caudal facet joint penetrates the opposing superior articular facet of the next vertebra, and FV in this situation may induce a problem leading to subsequent revision surgery for extended fixation.

In the review of patient charts, no serious complications such as neurovascular injuries, persistent postoperative palsy, or deep infection necessitating screw removal were documented. These results correspond to the majority of previous studies [14–22]. Among the complications identified in this review, postoperative C5 root palsy occurred in 5 patients (5.3 %) and 4 of these 5 patients (80 %) it was complicated with OPLL. Chen et al. [32] reported that C5 palsy after posterior cervical fixation occurred in 9 of 49 OPLL patients (18 %), and the incidence was further higher in patients with cervical lordosis and severe image changes. When surgery was performed for this cohort of patients, careful preoperative planning and postoperative observation are mandatory.

The strength of this study is the analysis of CT images obtained in the early postoperative period. Therefore, accurate assessment of the direction and location of screws as well as detection of intraoperative screw-related complications could be made. By contrast, the limitation of this study is that only the screw trajectory and length are analyzed to find the relationship between the rates of screw-related complications, while other factors such as morphologic characteristics also can influence the incidence. In addition, regarding the analysis of vascular complication, the collection of clinical data was solely based on the description of the operative record and the clinical findings in the patient’s chart, and special diagnostic modalities such as angiography and color-coded duplex sonography were not adopted. It has been also reported that the majority of intraoperative VA injuries are asymptomatic, and clinical manifestation can be delayed by several weeks in some cases [33]. Therefore, the analysis in the present study may have missed less evident vascular complications.

Although the present study showed the safety of our screwing procedure was acceptable, there were a considerable number of screws inserted with less optimal placement and trajectory. Whether use of intraoperative navigation or fluoroscopic control is able to improve the surgical consistency and reduce the incidence of the screw-related complication has yet to be examined. Moreover, complications such as FV and lateral mass fracture can affect the mechanical properties of the fixation, and thus further investigation for the subsequent clinical outcome is still required to substantially evaluate the safety and efficacy of our screwing procedure.

## Conclusions

The present study indicates that lateral mass screw fixation without intraoperative fluoroscopic images can be performed without serious complications. In the analysis of potential risk factors for violation of the VA foramen as well as FV during screw insertion, the former incidence was significantly related to the screw trajectory angle (lack of lateral angulation) in the axial plane, while the latter incidence was related to a poor screw trajectory angle in the sagittal plane. Moreover, the incidence of FV was highest at the C6 level. An understanding of these findings may help reduce the complication rate associated with cervical lateral mass screwing.

## References

[1] Gallie WE (1937). Skeletal traction in the treatment of fractures and dislocation of the cervical spine. Ann Surg.

[2] Roy-Camille R, Sailant G, Mazel C, Sherk H, Dunn H, Eismont F (1989). Internal fixation of the unstable cervical spine by a posterior osteosynthesis with plates and screws. The cervical spine.

[3] Anderson PA, Henley MB, Grady MS, Montesano PX, Winn HR (1991). Posterior cervical arthrodesis with AO reconstruction plates and bone graft. Spine.

[4] An H, Gordin R, Renner K (1991). Anatomic considerations for plate–screw fixation of the cervical spine. Spine.

[5] Jeanneret B, Magerl F, Ward E, Ward JC (1991). Posterior stabilization of the cervical spine with hook plates. Spine.

[6] Abumi K, Ito M, Taneichi H, Kaneda K (1994). Transpedicular screw fixation for traumatic lesion of the middle and lower cervical spine. Description of the technique and preliminary report. J Spinal Disord.

[7] Miyamoto H, Sumi M, Uno K (2009). Utility of modified transarticular screw in the middle and lower cervical spine as intermediate fixation in posterior long fusion surgery. J Neurosurg Spine.

[8] Kotani Y, McNulty PS, Abumi K, McAfee PC (1994). Biomechanical analysis of cervical stabilization systems: an assessment of transpedicular screw fixation in the cervical spine. Spine.

[9] Abumi K, Shono Y, Ito M, Taneichi H, Kotani Y, Kaneda K (2000). Complication of pedicle screw fixation in reconstructive surgery of the cervical spine. Spine.

[10] Neo M, Sakamoto T, Fujibayashi, Nakamura T (2005). The clinical risk of vertebral artery injury from cervical pedicle screws inserted in degenerative vertebrae. Spine.

[11] Yukawa Y, Kato F, Yoshihara H, Yanase M, Ito K (2006). Cervical pedicle screw fixation 100 cases of unstable cervical injuries: pedicle axis views obtained using fluoroscopy. J Neurosurg Spine.

[12] Miyamoto H, Uno K (2009). Cervical pedicle screw insertion using a computed tomography cutout technique. J Neurosurg Spine.

[13] Kotani Y, Abumi K, Ito M, Minami A (2003). Improved accuracy of computer-assisted cervical pedicle screw insertion. J Neurosurg Spine.

[14] Graham AW, Swank ML, Kinard RE, Lowery GL, Dials BE (1996). Posterior cervical arthrodesis and stabilization with a lateral mass plate. Clinical and computed tomographic evaluation of lateral mass screw placement and associated complications. Spine.

[15] Roche S, de Freitas DJ, Lenehan B, Street JT, McCabe JP (2006). Posterior cervical screw placement without image guidance: a safe and reliable practice. J Spinal Disord Tech.

[16] Heller JG, Slicox DH, Sutterlin CE (1995). Complications of posterior cervical plating. Spine.

[17] Huang RC, Girardi FP, Poynton AR, Cammisa FP (2003). Treatment of multilevel cervical spondylotic myeloradiculopathy with posterior decompression and fusion with lateral mass plate fixation and local bone graft. J Spinal Disord Tech.

[18] Deen HG, Brich BD, Wharen RE, Reimer R (2003). Lateral mass screw-rod fixation of the cervical spine: a prospective clinical series with 1-year follow-up. Spine J.

[19] Katonis P, Papadopoulos CA, Muffoletto A, Papagelopoulos PJ, Hadjipavlou AG (2004). Factors associated with good outcome using lateral mass plate fixation. Orthopedics..

[20] Sekhon LH (2005). Posterior cervical lateral mass screw fixation: analysis of 1026 consecutive screws in 143 patients. J Spinal Disord Tech..

[21] Wu JC, Huang WC, Chen YC, Shih YH, Cheng H (2008). Stabilization of subaxial cervical spines by lateral mass screw fixation with modified Magerl’s technique. Surg Neurol.

[22] Katonis P, Papadakis S, Galanakos S (2011). Lateral mass screw complications: analysis of 1662 screws. J Spinal Disord Tech..

[23] Heller JG, Carlson GD, Abitbol JJ, Garfin SR (1991). Anatomic comparison of the Roy-Camille and Magerl techniques for screw placement in the lower cervical spine. Spine.

[24] Pait TG, McAlister PV, Kaufman HH (1995). Quadrant anatomy of the articular pillars (lateral cervical mass) of the cervical spine. J Neurosurg.

[25] Ebraheim NA, Xu R, Challgren E, Yeasting RA (1997). Quantitative anatomy of the cervical facet and the posterior projection of its inferior facet. J Spinal Disord.

[26] Ebraheim NA, Klausner T, Xu R, Yeasting RA (1998). Safe lateral-mass screw lengths in the Roy-Camille and Magerl techniques. An anatomic study. Spine.

[27] Barrey C, Mertens P, Jund J, Cotton F, Perrin G (2005). Quantitative anatomic evaluation of cervical lateral mass fixation with a comparison of the Roy-Camille and the Magerl screw techniques. Spine.

[28] Abdullah KG, Steinmetz MP, Mroz TE (2009). Morphometric and volumetric analysis of the lateral masses of the lower cervical spine. Spine.

[29] Seybold EA, Baker JA, Criscitiello AA, Ordway NR, Park CK, Connolly PJ (1999). Characteristics of unicortical and bicortical lateral mass screws in the cervical spine. Spine.

[30] Ebraheim NA, Hoeflinger MJ, Salpietro B, Chung SY, Jackson WT (1991). Anatomic considerations in posterior plating of the cervical spine. J Orthop Trauma.

[31] Ebraheim NA, Xu R, Yeasting RA (1996). The location of the vertebral artery foramen and its relation to posterior lateral mass screw fixation. Spine.

[32] Chen Y, Chen D, Wang X, Guo Y, He Z (2007). C5 palsy after laminectomy and posterior cervical fixation for ossification of posterior longitudinal ligament. J Spinal Disord Tech.

[33] Wright NM, Lauryssen C (1998). Vertebral artery injury in C1–2 transarticular screw fixation: results of a surgery of the AANS/CNS section on disorders of the spine and peripheral nerves. J Neurosurg.

